# Metabolic diversity analysis and genome wide assessment of oxalate accumulation in the leaves of rice (*Oryza sativa*) cultivars

**DOI:** 10.5511/plantbiotechnology.23.1025a

**Published:** 2024-03-25

**Authors:** Atsuko Miyagi, Nobuhiro Tanaka, Matthew Shenton, Kaworu Ebana, Satoshi Ohkubo, Shunsuke Adachi, Taiichiro Ookawa, Maki Kawai-Yamada

**Affiliations:** 1 Faculty of Agriculture, Yamagata University, 1-23 Wakaba-machi, Tsuruoka, Yamagata 997-8555, Japan; 2 Institute of Crop Science, National Agriculture and Food Research Organization, 2-1-2 Kannondai, Tsukuba, Ibaraki 305-8518, Japan; 3 Research Center of Genetic Resources, National Agriculture and Food Research Organization, 2-1-2 Kannondai, Tsukuba, Ibaraki 305-8518, Japan; 4 Graduate School of Agriculture, Tokyo University of Agriculture and Technology, 5-8 Saiwai-cho, Fuchu, Tokyo 183-8509, Japan; 5 Graduate School of Science and Engineering, Saitama University, 255 Shimo-Okubo, Sakuraku, Saitama, Saitama 338-8570, Japan

**Keywords:** CE-MS, GWAS, *Oryza sativa*, oxalate

## Abstract

Soluble oxalate accumulates in rice leaves, and it causes mineral deficiency and urinary syndrome in livestock that consume the leaves. In our previous study, we found that the oxalate content was higher in the leaves of Koshihikari (*japonica* type cultivar) than in those of Takanari (*indica* type cultivar). This difference was seen even when the two cultivars were grown under a high CO_2_ concentration, which inhibits oxalate synthesis via photorespiration, suggesting that the difference resulted from genetic factors rather than environmental factors. To clarify whether genetic factors affect the oxalate content of rice leaves, we measured the contents of oxalate and oxalate-related organic acids in the leaves of various rice cultivars the Rice Core Collection (WRC) and Japan Rice Core Collection (JRC) by capillary electrophoresis-mass spectrometry. Results showed that *japonica* type cultivars tended to accumulate more oxalate than *aus* or *indica* type cultivars. Correlation analysis revealed a positive correlation between oxalate accumulation and the citrate content, suggesting that the isocitrate pathway is involved in oxalate accumulation. On the other hand, a genome-wide association study for the oxalate content of the WRC and JRC cultivars did not reveal significant loci directly related to oxalate accumulation. This indicates that the combination of various loci may affect the oxalate contents of rice leaves.

## Introduction

Rice straw, i.e., dried rice leaves and stems, can be used as a feed for livestock. In Japan, approximately one-fourth of the total rice straw used for animal is imported. However, increasing prices and declining quality due to molds and/or feces have become issues with imported straw. Moreover, more than 90% of domestic rice straw is disposed of by plowing or incineration. Thus, promotion of the use of domestic rice straw for animal feed has become an agricultural issue in Japan.

Soluble oxalate is often accumulated in rice leaves. In plants, oxalate plays important roles in the defense against animals (Miyagi et al. 2013a), chelate formation with metal ions, such as aluminium (Ma et al. 2001; Miyagi et al. 2013b), signaling in aging and wounding as a source of H_2_O_2_ (Davoine et al. 2001; Le Deunff et al. 2004), and control of calcium levels (Franceschi and Nakata 2005), among others. However, oxalate is also a toxic molecule that leads to mineral deficiency and urinary diseases in humans and vertebrates (Jackson 1977; Reig et al. 1990). To promote the use of domestic rice straw for animal feed, it is important to reduce the oxalate content of rice leaves.

There are three reported oxalate biosynthesis pathways in plants: the isocitrate, glycolate, and ascorbate pathways (Franceschi and Nakata 2005). In the isocitrate pathway, isocitrate is catalyzed to glyoxylate and succinate by isocitrate lyase in the glyoxylate cycle. In the glycolate pathway, which is part of the photorespiration pathway, glycolate is catalyzed to glyoxylate by glycolate oxidase. The glyoxylate produced from isocitrate and glycolate is then oxidized to oxalate. In the ascorbate pathway, ascorbate is oxidized to dehydroascorbate, which is subsequently catalyzed to oxalate via several steps. However, it remains unknown which pathway(s) or enzyme(s) contribute to the oxalate accumulation in rice leaves. In our previous study, we found that the oxalate content of Koshihikari (*japonica* type cultivar) leaves was higher than that of Takanari (*indica* type cultivar) leaves. The difference in the oxalate content between the two rice cultivars was seen even when they were grown under elevated CO_2_ levels and/or temperatures, suggesting that the difference in the oxalate content results from genetic factors rather than environmental factors (Miyagi et al. 2019). It has been reported that ion beam irradiation changes the oxalate content of rice (Koshihikari) populations. A metabolome analysis of these rice leaves revealed that the isocitrate pathway or ascorbate pathway was involved in the oxalate synthesis in the rice leaves (Miyagi et al. 2020).

In the present study, we attempted to identify factors that directly affect oxalate accumulation in rice by focusing on the variations in the oxalate content among rice cultivars. Namely, we first measured the contents of oxalate and oxalate-related organic acids in the leaves of 107 rice cultivars in the World Rice Core Collection (WRC) and Japanese Rice Core Collection (JRC) by capillary electrophoresis-mass spectrometry (CE-MS). Subsequently, we performed a genome-wide association study (GWAS) for the oxalate contents and single-nucleotide polymorphisms (SNPs) to identify loci involved in oxalate biosynthesis in rice leaves.

## Materials and methods

### Plant materials

The seeds of rice cultivars (WRC: 57 cultivars; JRC: 50 cultivars; Supplementary Data S1) were provided by the Genebank Project, National Agriculture and Food Research Organization (NARO), Japan. Field experiments were conducted in a paddy field at the University Farm of Tokyo University of Agriculture and Technology (35°41′N, 139°29′E) in Fuchu-city, Tokyo, Japan, according to Ohkubo et al. (2020) and Honda et al. (2021). The seeds were sown in nursery boxes filled with artificial soil (Shinano soil, Ohata Seed, Saitama, Japan) in May 2018 (WRC), 2020, or 2021 (JRC), and seedlings were grown until the 4th leaf stage in a greenhouse. Subsequently, 21-day-old seedlings were transplanted in an experimental paddy field at a density of one seedling per hill, and at a spacing of 15 cm between hills and 30 cm between rows. A basal dressing of inorganic fertilizer supplying 30 kg N, 60 kg P, and 60 kg K ha^−1^ was applied. One-third of the total N was applied as ammonium sulfate, and the other two-thirds as slow-release urea (LP-50 & LPS-100; JCAM Agri Co., Ltd., Tokyo, Japan). No topdressing was applied, and the level of standing water was maintained at 2 to 4 cm above the soil. For oxalate measurements by CE-MS, from July to September 2018 (WRC), 2020, or 2021 (JRC), flag leaves collected after heading were immediately frozen in liquid nitrogen, and stored at −80°C.

### Measurement of organic acids by CE-MS

Oxalate and other organic acids were extracted by the methods of Miyagi et al. (2020) with minor modifications. Briefly, frozen leaves (approximately 30 mg) were ground and homogenized using a Shake Master Neo Ver. 1.0 (Bio Medical Science, Tokyo, Japan). Metabolites were extracted with 50% methanol containing 50 µM piperazine-1,4-bis(2-ethanesulfonic acid) (PIPES) as an internal standard. After the first centrifugation at 22,000×g for 5 min at 4°C, the supernatant was transferred to a 3 kDa cut-off filter (Merck, Billerica, MA, USA). After centrifugation again at 14,000×g for 30 min at 4°C, the supernatants were collected and used for the CE-MS analysis.

The contents of the organic acids were measured using a CE-QQQ-MS system (CE: 7100; MS: G6420A; Agilent Technologies, Santa Clara, CA, USA) in the multi-reaction monitoring (MRM) mode according to the methods of Miyagi et al. (2020). A DB-WAX capillary (polyethylene glycol-coated, 100 cm×50 µm in diameter) with 20 mM ammonium acetate (pH 8.5) as a running buffer was used with −25 kV applied voltage in the negative ion mode. For MS stabilization, 5 mM ammonium acetate in 50% (v/v) methanol was used as a sheath solution, and applied to the capillary at 10 µl min^−1^ using an isocratic HPLC pump (Agilent 1200 series) equipped with a 1 : 100 splitter. The capillary voltage (−3,500 V) and the drying nitrogen gas (at 320°C and a flow rate of 8 l min^−1^) were kept constant for approximately 30 min during each electrophoresis run. The quantitative accuracy was determined using known concentrations of standard reference compounds with Agilent MassHunter Software.

Bivariate correlation analysis between oxalate and other metabolites was performed based on the Pearson’s correlation coefficient using the IBM SPSS software package v27.0 (IBM, Armonk, NY, USA) according to the methods of Miyagi et al. (2013a).

### GWAS for the oxalate content

A GWAS was performed employing mixed linear models (MLM) in R using scripts from the GENESIS R package (Gogarten et al. 2019). Manhattan plots and QQ plots were drawn using the qqman package (Turner 2018).

## Results

### Oxalate contents of the leaves of rice cultivars

**Figure 1:**
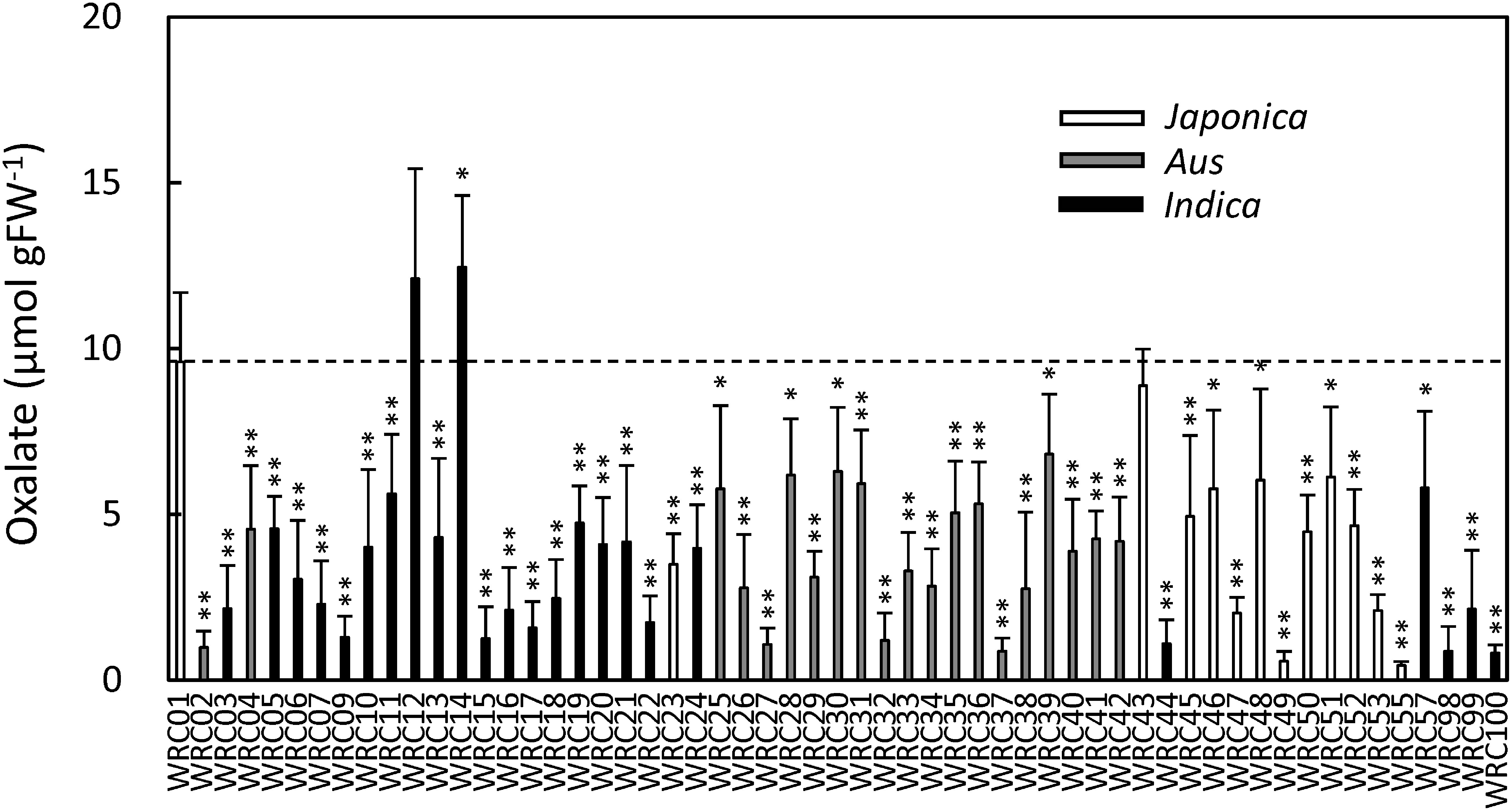
Figure 1. Oxalate content of the flag leaves of WRC cultivars grown at the paddy field of Tokyo University of Agriculture and Technology in 2018. Dot line represents the content of WRC 01 (Nipponbare). The student’s *t*-test was performed based on WRC 01. *n*=6; bars: standard deviation; *: *p*<0.05; **: *p*<0.01.

**Figure 2:**
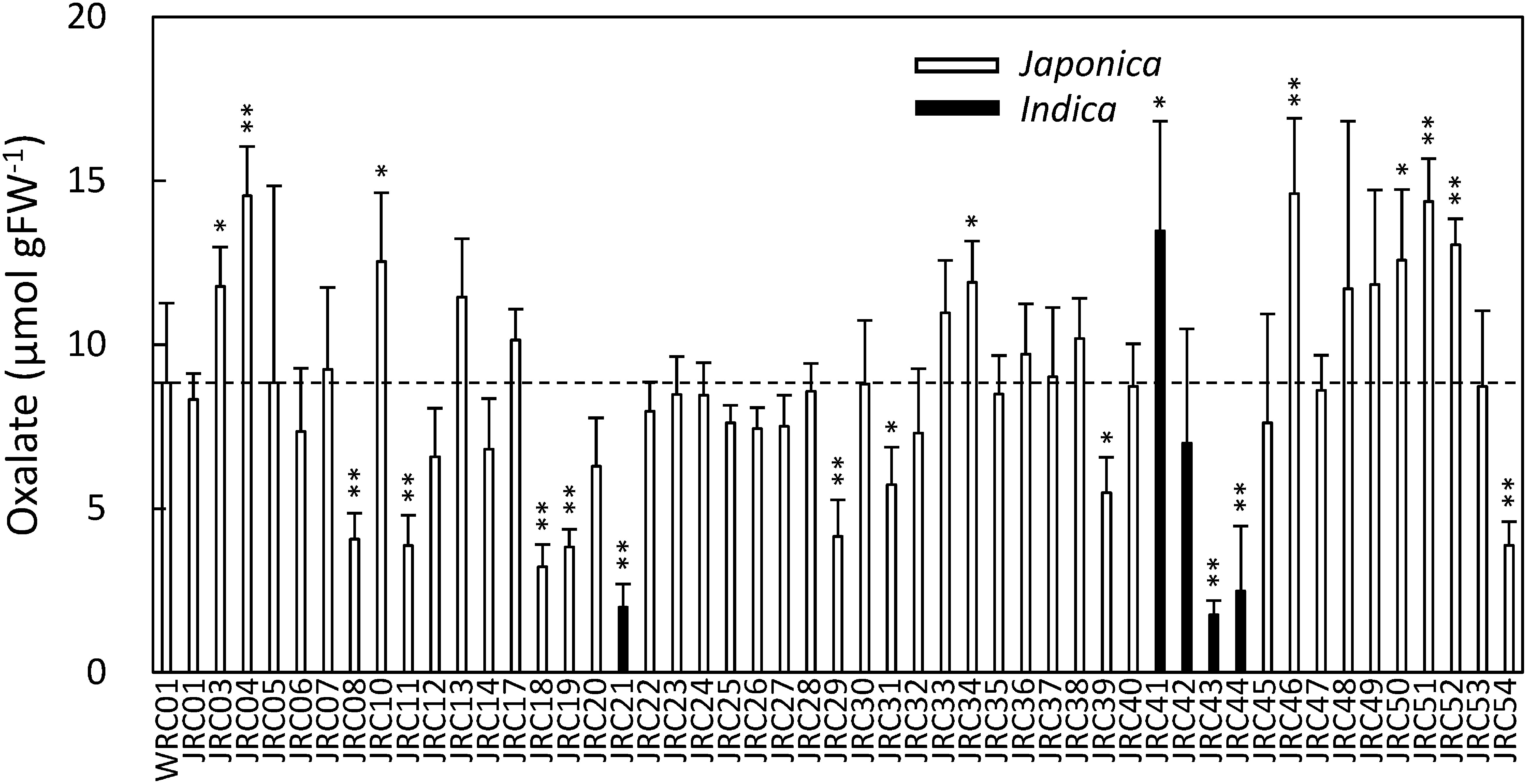
Figure 2. Oxalate content of the flag leaves of WRC 01 (Nipponbare) and JRC cultivars grown at the paddy field of Tokyo University of Agriculture and Technology in 2020. Only JRC 01 (Gaisen mochi) and 08 (Okka modoshi) was grown and harvested in 2021. Dot line represents the content of WRC 01. The student’s *t*-test was performed based on WRC 01. *n*=6; bars: standard deviation; *: *p*<0.05; **: *p*<0.01.

**Figure 3:**
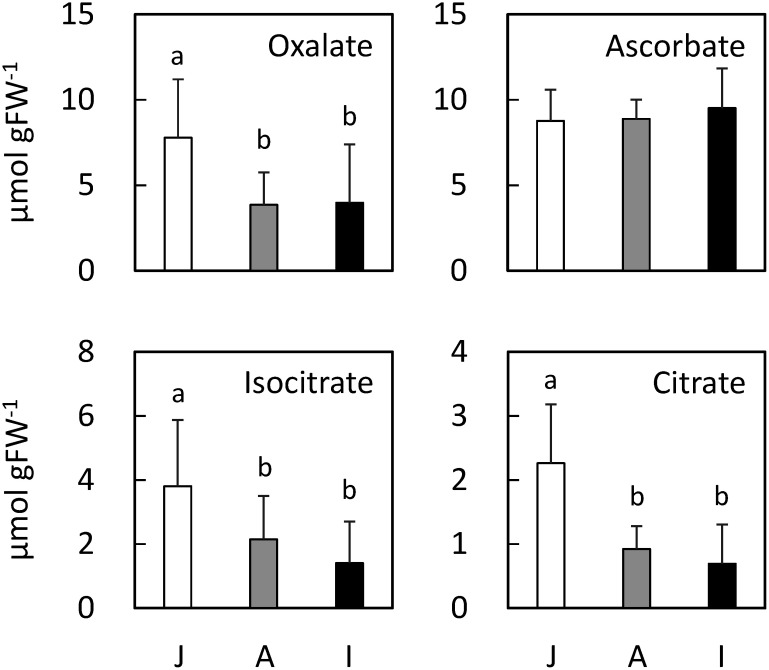
Figure 3. Average of oxalate content among the *japonica* (*n*=58), *aus* (*n*=20), or *indica* (*n*=29) type cultivars. J: *japonica* type cultivars; A: *aus* type cultivars; I: *indica* type cultivars; Bars: standard deviation; lowercase letters represent Tukey’s HSD (*p*<0.05).

The oxalate contents of the flag leaves of the 57 rice cultivars in the WRC were quantified by CE-MS. The WRC cultivars were genetically classified according to the methods of Tanaka et al. (2020a) instead of NARO’s morphological classification (See Supplementary Data S1). The results revealed variations in the oxalate content among the cultivars; the oxalate content was highest in WRC 14 (IR 58) and lowest in WRC 55 (Tupa 729), and there was a more than 20-fold difference between the two cultivars (Figure 1). We also measured the oxalate content of the flag leaves of 50 rice cultivars in the JRC. The JRC cultivars were also genetically classified according to the methods of Tanaka et al. (2020b) instead of NARO’s morphological classification (See Supplementary Data S1). The results revealed variations in the oxalate content among the JRC cultivars (Figure 2); the oxalate content was highest in JRC 46 (Fukoku), and lowest in JRC 43 (Akamai), and there was an 8.3-fold difference between the two cultivars. To examine the influence of genetic variations on the accumulation of oxalate, we calculated the average oxalate content of the *japonica* (*n*=58), *aus* (*n*=20), or *indica* (*n*=29) type cultivars from both the WRC and JRC cultivars (*n*=107). The results showed that the average oxalate content of *japonica* type cultivars was approximately two times more than that of the *aus* or *indica* type cultivars (Figure 3).

**Figure 4:**
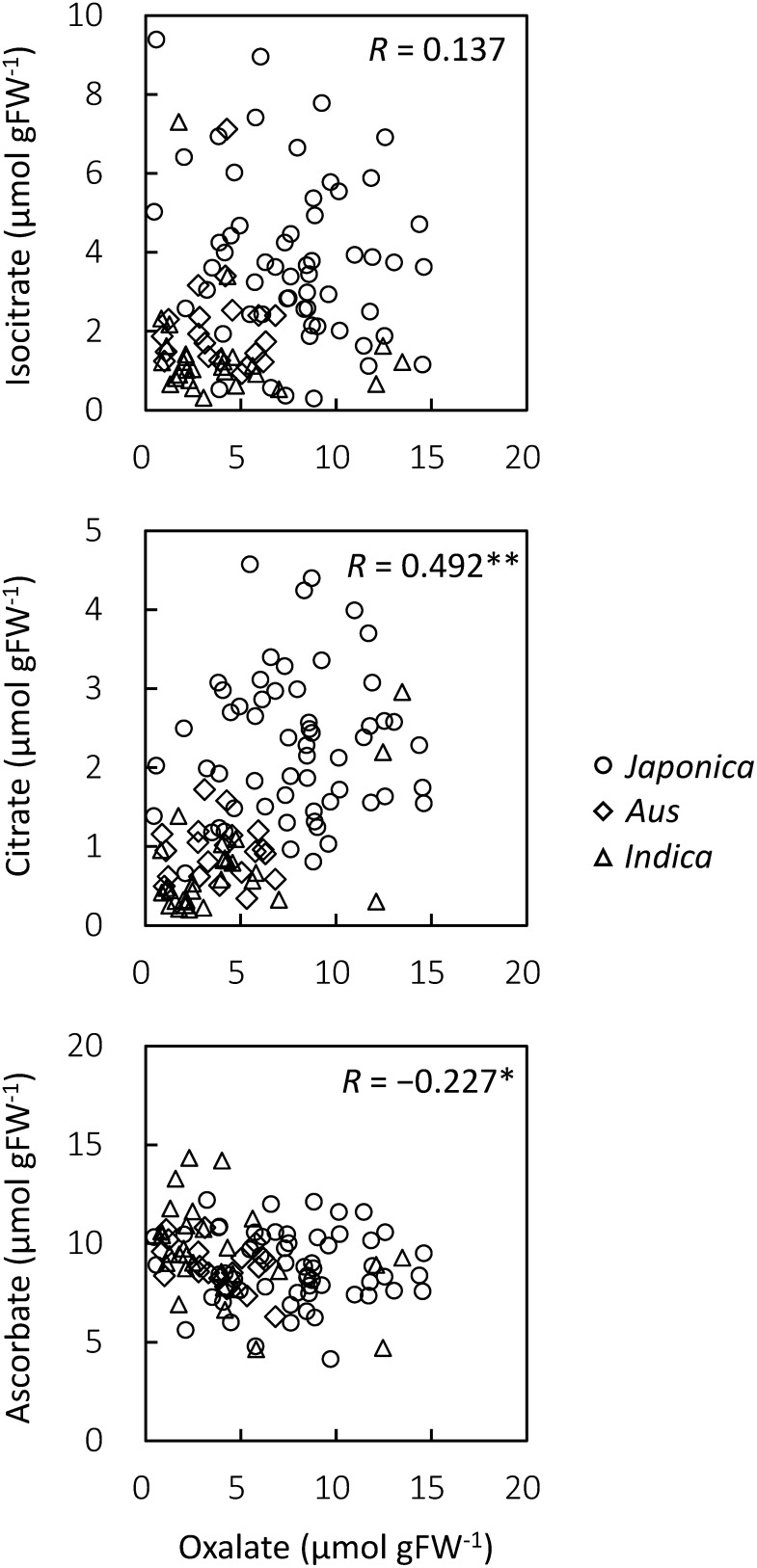
Figure 4. Correlation analysis between oxalate and other organic acids. *R*: Pearson’s correlation coefficient; *: *p*<0.05; **: *p*<0.01.

To evaluate the effect of other organic acids on the oxalate content, we also measured the contents of organic acids involved in oxalate biosynthesis by CE-MS, and preformed bivariate correlation analyses between oxalate and the organic acids. Similar to oxalate, citrate and isocitrate, which are involved in the isocitrate pathway, were also accumulated more in the *japonica* type cultivars than in the *aus* and *indica* type cultivars (Figure 3). In contrast, the ascorbate content did not differ significantly between the *japonica*, *aus*, and *indica* type cultivars. Bivariate correlation analysis revealed that citrate content was positively correlated with the oxalate content, whereas no correlation was seen between the isocitrate and oxalate contents (Figure 4). The contents of other organic acids involved in the TCA or glyoxylate cycle, with exception of 2OG, tented to positively correlated with the oxalate content (Supplementary Data S2). The 2OG content showed a negatively correlation with the oxalate content. A weak negative correlation was seen between ascorbate and oxalate (Figure 4). Glycolate and glyoxylate, which are involved in the glycolate pathway, were not detected.

### GWAS for the oxalate content of the leaves of various rice cultivars

**Figure 5:**
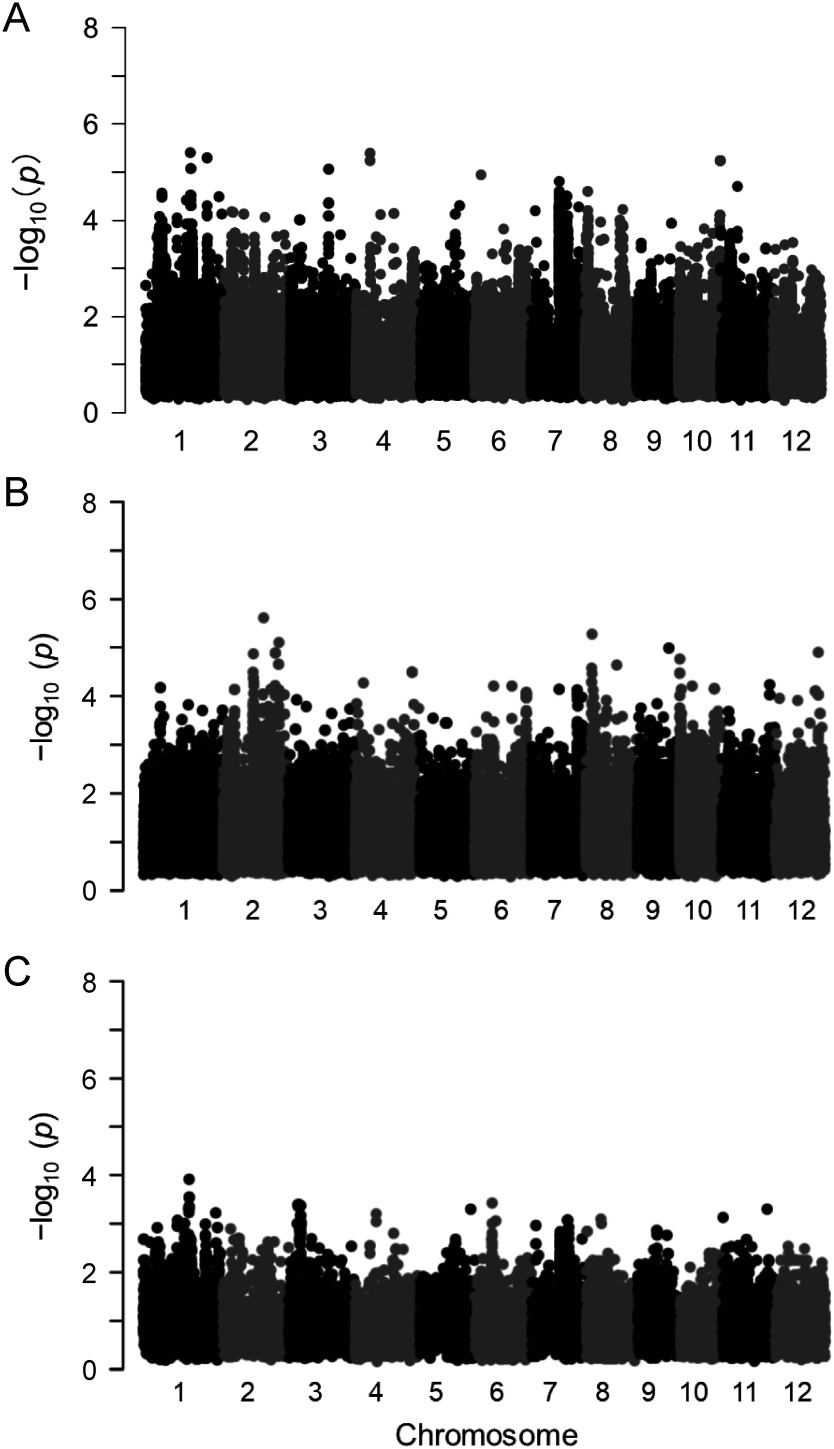
Figure 5. Manhattan plots of the GWAS for the oxalate content and SNPs in all chromosomes among the 107 rice cultivars in the WRC or JRC. A: WRC and JRC cultivars; B: WRC cultivars; C: JRC cultivars.

**Figure 6:**
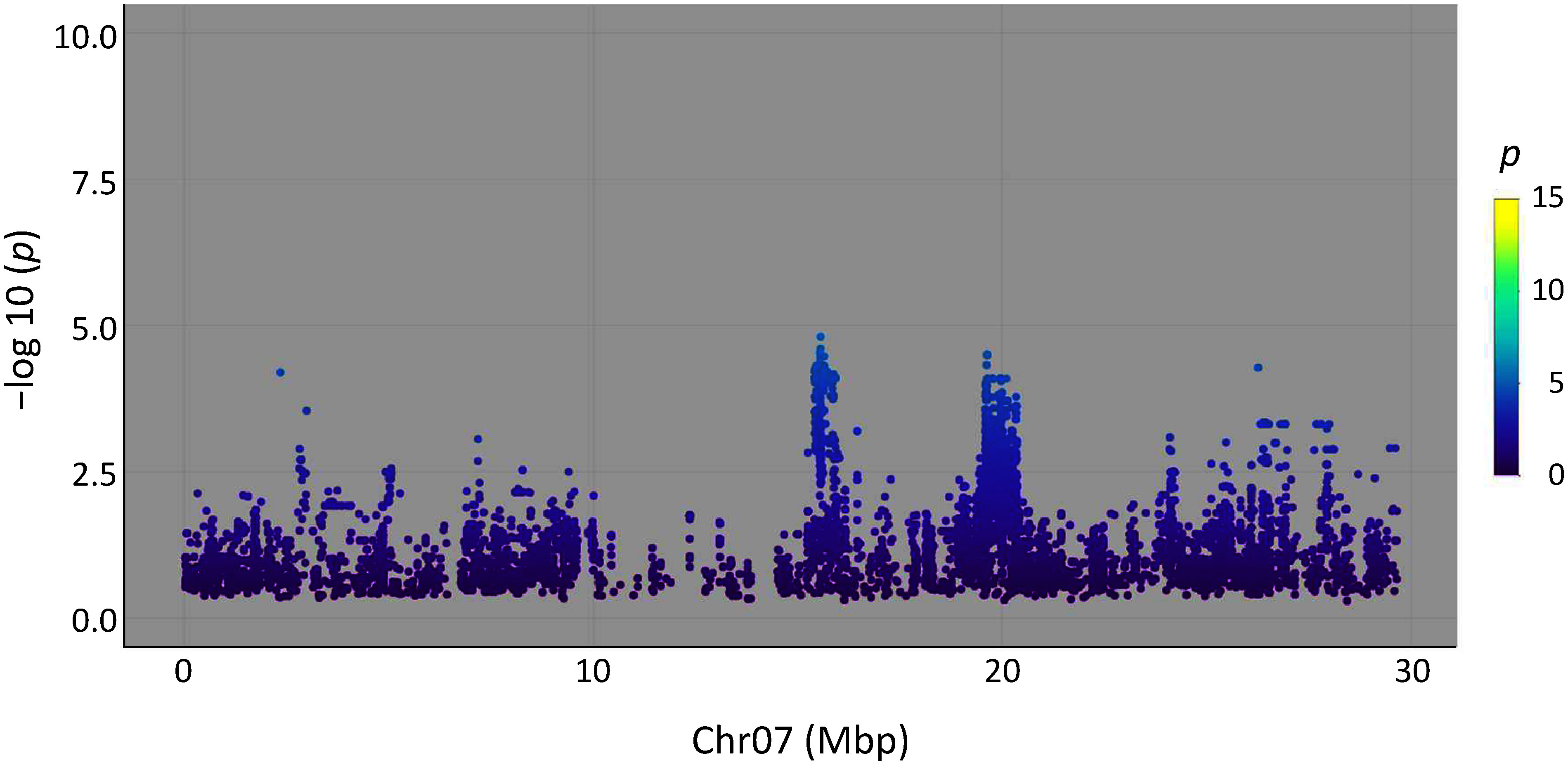
Figure 6. Manhattan plots of the GWAS using oxalate content and SNPs in chromosome 7 among the 107 rice cultivars in the WRC or JRC.

The measurements of the oxalate content revealed that the oxalate content of rice leaves differed significantly among rice cultivars in the WRC or JRC. Recently, the whole genome sequences of these rice cultivars have been decoded (Tanaka et al. 2020a, 2020b). Thus, we performed a GWAS using the datasets on the oxalate content (average values of each cultivar) and SNPs in the chromosomes of rice cultivars to identify loci involved in oxalate accumulation in rice leaves. Contrary to our expectations, the results identified no significant locus that significantly affects the oxalate content of rice leaves (Figure 5A). We reanalyzed the GWAS data for the WRC and JRC cultivars separately, but the results again showed no locus that significantly affects the oxalate content (Figure 5B, C). In the GWAS results for both the WRC and JRC cultivars (Figure 5A), a relatively high peak was observed on chromosome 7 (Figure 6). A GWAS was also performed using the median values of the oxalate content of each cultivar, and the same results were obtained (data not shown).

## Discussion

### Variations in the oxalate content among the rice cultivars

To evaluate the relationship between the oxalate content and genetic diversity, we compared the oxalate content of the flag leaves of the WRC and JRC cultivars. In the WRC cultivars, the oxalate content of the *aus* and *indica* type cultivars tended to be lower than that of the *japonica* type cultivars. In a previous study, the oxalate content of Koshihikari (*japonica* type cultivar) leaves was higher than that of Takanari (*indica* type cultivar) leaves (Miyagi et al. 2019). Taichung 65 (*japonica* type cultivar) leaves also accumulated more oxalate than IR 36 and 68 (*indica* type cultivars) leaves (Miyagi et al. 2022). A genomic analysis has revealed that *japonica* type cultivars may have derived from a group of wild rice *Oryza rufipogon*, and that *indica* type cultivars may have resulted from the crossbreeding of ancient *japonica* type cultivars and another group of *O. rufipogon* (Huang et al. 2012). This indicates that the oxalate content may have changed due to some genetic mutation(s) that occurred during the course of rice domestication. However, it remains unknown why the oxalate content of the leaves of *japonica* type cultivars is higher than that of the leaves of *aus* and *indica* cultivars. It has been reported that Koshihikari is more tolerant to ozone exposure than Takanari (Sakoda et al. 2022). Oxalate is derived from the oxidation of glyoxylate or ascorbate. The glycolate and ascorbate pathway, which are oxalate synthesis pathway, is involved in photosynthesis. Glycolate is produced by photorespiration in the case that excessive O_2_ produced via photosynthesis is fixed to RuBP by RuBisCO. Ascorbate is synthesized in plastids during light period, and it oxidized due to eliminate reactive oxygen species (ROS). ROS is often occurred by high light and low temperature. *Japonica* type cultivars tend to be adaptive to low temperature than *aus* and *indica* type cultivars (Fukuda and Terao 2015; Ohsumi et al. 2012). These may result in *japonica* type cultivars accumulating more oxalate than *aus* and *indica* type cultivars. Thus, oxalate synthesis may play an important role in decreasing some kind of oxidative stress in the leaves of *japonica* type cultivars. Further studies are needed to investigate this hypothesis.

Oxalate content is known to be robust to environmental constraints because it is an end product accumulated in vacuole except for aging or wounding period. It had been reported that the robustness of strong correlation between oxalate and other organic acids, especially citrate, is kept in *Rumex* plants: nutrition or CO_2_ supply, light condition, low temperature, and Al^3+^ treatment (Miyagi et al. 2010a, 2010b, 2011, 2013b). A positive correlation between oxalate and citrate was also shown in the present study. It is indicated that citrate would be an important source that affects oxalate synthesis of rice leaves via isocitrate pathway.

### Genetic mechanisms of oxalate accumulation

Oxalate is synthesized and accumulated mainly in leaves according to the leaf growth (Miyagi et al. 2010a). On the other hand, oxalate degradation is occurred in the case of wounding or senescence. Oxalate efflux from roots had been reported in polygonaceous species such as *Fagopyrum esculentum* Moench. (buckwheat) grown under a presence of aluminium ions in acidic soil. However, the roots accumulate oxalate less than the leaves (Ma et al. 1998), and the levels of oxalate efflux is extremely low relative to the oxalate content in the roots (Zheng et al. 1998). These suggests that oxalate content is dependent on the rate of oxalate synthesis. Thus, we focused on the oxalate synthesis pathway in rice leaves.

The use of a genetic approach to examine the oxalate synthesis pathway has been reported, but they were unable to identify any gene that directly affects the oxalate content (Yu et al. 2010). On the other hand, a metabolomic analysis revealed that treatment with itaconate, which inhibits isocitrate lyase activity, drastically reduced the oxalate content of the leaves of *Rumex obtusifolius* L. (bitter dock, Polygonaceae) to less than one-tenth of the normal level, suggesting that the isocitrate pathway contributes to oxalate accumulation in *R. obtusifolius* (Miyagi et al. 2013c). In low-oxalate strains of Koshihikari obtained from double ion beam-irradiated seeds, the contents of citrate and ascorbate tended to be correlated to the oxalate content. Thus, we expected that isocitrate and/or ascorbate pathways would be correlated with the genes (especially the gene for isocitrate lyase) or loci that may be involved in oxalate synthesis and the oxalate contents. We first expected that isocitrate lyase (ICL) gene, which is located on the long arm region of the seventh chromosome, would be detected. However, a weak correlation was found in the seventh chromosome, and isocitrate lyase and other related genes are not encoded in this region. A positive reason why no gene was identified is that the combination of various genes involves the oxalate content of rice leaves. On the other hand, the *p*-values of some SNPs in Figure 5B is lower than those of Figure 5A or C, although none exceeded the general significance level of 5×10e^−8^. *Japonica* type cultivars are divided to two genetic groups J-1 and J-2 type as shown in the Supplementary data S1 (Tanaka et al. 2020b). *Japonica* type cultivars used in the Figure 5B (WRC) is used only J-1 type, although both J-1 and J-2 type were included in the Figure 5A (WRC and JRC) and 5C (JRC). This differences would be reflected the results of GWAS, suggesting that genetic characteristics and oxalate contents of J-2 type cultivars would contribute to gain the *p*-values of SNPs.

It has been reported that the oxalate content of rice leaves is also affected by environmental factors. Oxalate accumulation is induced more by nitrate content than by ammonium in the soil (Tian et al. 2008). In the examined rice cultivars, nitrate reductase activities affect oxalate contents, but there was no significant correlation between oxalate and the nitrate reductase gene in the GWAS. A high CO_2_ concentration reduces the oxalate content of some plant, such as Koshihikari; however, the oxalate content was not affected by a high CO_2_ concentration in Takanari (Miyagi et al. 2019). A high CO_2_ concentration inhibits photorespiration, and subsequently reduce oxalate accumulation via the glycolate pathway. These results suggest that the variations in the oxalate content of leaves of rice cultivars in the WRC and JRC likely result from genetic factors rather than environmental factors.

In conclusion, we found that the leaves of *japonica* type cultivars tended to accumulate more oxalate than those of *aus* and *indica* type cultivars, suggesting that the oxalate content may have changed over the course of rice domestication. However, the GWAS results did not reveal any loci that affect oxalate accumulation. Another approach to identify loci that contribute to oxalate accumulation is needed to clarify the mechanisms of oxalate accumulation, and the reason why *japonica* type rice cultivars came to accumulate more oxalate than *aus* and *indica* type cultivars over the course of rice domestication.

## Abbreviations

CE-MS: capillary electrophoresis-mass spectrometry
GWAS: genome-wide association study
JRC: Japan Rice Core Collection
SNP: single nucleotide polymorphism
TCA: tricarboxylic acid
2OG: 2-oxoglutarate
WRC: World Rice Core Collection

## Description of Supplementary Files

Supplementary Data S1. The dataset of rice cultivars used in the present study.

Supplementary Data S2. Correlation analysis between oxalate and its related organic acids of the flag leaves of the WRC and JRC cultivars.
