# Supplementary material for: Metabolic diversity analysis and genome wide assessment of oxalate accumulation in the leaves of rice (Oryza sativa) cultivars
Source: Plant Biotechnol (Tokyo). 2024 Mar 25;41(1):1–7. doi: 10.5511/plantbiotechnology.23.1025a (PMC11500591; doi:10.5511/plantbiotechnology.23.1025a)
Supplement: Supplementary Data [file plantbiotechnology-41-1-23.1025a-s001.pdf]

Metabolite	*R*	*P*
Citrate	0.492	0.000
Aconitate	0.243	0.012
Isocitrate	0.137	0.160
2-oxoglutarate	−0.314	0.001
Succinate	0.371	0.000
Fumarate	0.179	0.065
Malate	0.485	0.000
Ascorbate	−0.227	0.019

Supplementary Data S2. Correlation analysis between oxalate and its related organic acids of the flag leaves of the WRC and JRC cultivars. R: Pearson's correlation coefficient.
